# Risk factors for harmful alcohol consumption among employees and intervention strategies used in the workplace: current state of knowledge based on a literature review

**DOI:** 10.3389/fpubh.2025.1685325

**Published:** 2026-01-05

**Authors:** Marta Giezek, Andriej Szpakow, Beata Mintus, Beata Karakiewicz

**Affiliations:** 1 Department of Social Medicine and Public Health, Chair of Social Medicine, Pomeranian Medical University in Szczecin, Szczecin, Poland; 2 Department of Nursing, International Academy of Applied Sciences in Łomża, Łomża, Poland; 3 Institute of Sociology, University of Szczecin, Szczecin, Poland

**Keywords:** alcohol use, employees, intervention, occupational health, prevention, risk factors, workplace

## Abstract

**Introduction:**

Harmful alcohol consumption among employees is a significant public health problem, leading to adverse health and social outcomes, as well as impaired occupational functioning. Identifying risk factors and effective prevention strategies is crucial to address this issue in the workplace. The aim of this study was to analyze the main determinants of harmful alcohol use among employees and to assess the effectiveness of interventions in occupational settings.

**Methods:**

A literature review was conducted on studies published between 2020 and 2024. The PubMed, Scopus, and Web of Science databases were searched for international studies on workplace alcohol consumption and intervention strategies. Key risk factors, workplace interventions, and their effectiveness were analyzed. Inclusion criteria included peer-reviewed articles published in English, a focus on employees or the occupational context, and a clear analysis of risk factors or interventions addressing harmful alcohol consumption. Titles and abstracts were independently reviewed by two researchers, and any discrepancies were resolved by a third. Data on risk factors, types of interventions, target populations, and outcome measures were extracted and summarized in tables.

**Results:**

Key risk factors identified comprise high job strain, workplace-related stress, low social support, job insecurity, unfavorable organizational culture, and shift work. Harmful alcohol use was associated with increased risk of workplace accidents, absenteeism, and diminished productivity. Effective preventive approaches included the implementation of digital interventions (e.g., mobile health applications, online brief interventions), involvement of management in educational campaigns, organizational policy changes, and multi-level, sustained action plans. Interventions targeting both individual and organizational levels demonstrated the greatest potential for sustainable impact.

**Conclusion:**

Reducing harmful alcohol use in the workplace requires a comprehensive, multi-level approach tailored to organizational context and providing long-term employee support. Continued evaluation and adaptation of intervention strategies to specific workplace environments are recommended.

## 1 Introduction

Alcohol is a legal, most widely used, readily available and, at the same time, the cheapest psychoactive substance that causes serious health, social, and economic consequences (1, 2).

In the literature on alcohol use, numerous terms and definitions are employed that—although precise in their original contexts—are often applied differently in practice by researchers, policymakers, the media, law enforcement, and healthcare professionals. To encompass the broad spectrum of phenomena related to alcohol consumption, this article adopts the umbrella terms “substance use” and “problematic use” or “harmful use” in a general sense, in accordance with current terminological recommendations. These terms allow for the description of both patterns of alcohol consumption and their associated health and social consequences, without unduly narrowing the scope of meaning.

In contexts that require definitional precision—such as occupational medicine, screening procedures, or references to established diagnostic classification—terms with strictly defined meanings must be used. In particular, the category alcohol use disorders (AUD) should not be employed as an overarching label for the entire spectrum of alcohol use. In the Diagnostic and Statistical Manual of Mental Disorders, Fifth Edition classification, AUD constitutes a specific diagnostic entity comprising three severity levels (mild, moderate, and severe), defined by a set of symptoms grouped into four domains: impaired control, social impairment, risky use, and pharmacological criteria, such as tolerance and withdrawal symptoms (3).

In accordance with the terminology used by the WHO, some of the data cited refer to the harmful use of alcohol rather than to AUD. In this context, the WHO estimates that in 2016 the harmful use of alcohol accounted for approximately 3 million deaths worldwide (5.3% of all deaths) and 132.6 million disability-adjusted life years (DALYs)^1^—that is, 5.1% of all DALYs that year (4). By contrast, prevalence data for AUD are reported separately: globally, an estimated 237 million men and 46 million women meet the diagnostic criteria for AUD, with the highest prevalence observed in the European region and the region of the Americas (4). For these reasons, this article maintains semantic and definitional consistency by applying terminology that aligns with the context of the data sources, classifications, and analytical objectives.

The development of harmful alcohol use can lead to permanent, irreversible changes in both physical and mental health, and make it difficult to establish and maintain interpersonal relationships and achieve life goals (5). In addition, the economic toll of alcohol is huge—according to studies, the economic cost associated with alcohol consumption exceeds 1% of gross domestic product in middle- and high-income countries (6).

There are three basic drinking patterns that generate risks and require appropriate interventions and changes. The problematic behaviors associated with alcohol consumption are considered to be:

1. Risky drinking—the one-off intake of excessive amounts of alcohol over a short period of time, which does not yet cause current negative consequences. However, if this way of drinking becomes entrenched, serious health and social consequences can be expected.
2. Harmful drinking—regular or episodic consumption of alcohol that already leads to damage to physical, mental, and social health, with no symptoms of dependence yet present.
3. Alcohol dependence—the consequence of long-term harmful drinking when drinking becomes an overriding life activity (7).^2^

It should be emphasized, however, that there is not always a continuous progression from risky drinking to harmful drinking and, ultimately, to alcohol dependence. Even occasional risky alcohol consumption can lead to negative health and social consequences and create a significant burden on the healthcare system. An intoxicated individual—even occasionally—may also cause harm to others, for instance, through workplace absenteeism, conflicts with co-workers, or occupational accidents.

Alcohol affects the central nervous system, causing significant changes in mood, perception, or behavioral control. These effects depend on the blood alcohol concentration—levels above 0.5‰ already lead to clearly negative effects such as impaired judgment, impaired memory and coordination, decreased alertness or deterioration of self-control. Exceeding this threshold is also associated with an increased risk of legal liability (8). In Polish law, the level of alcohol determines the qualification of the act—from 0.2 to 0.5‰ is considered a misdemeanour, above 0.5‰ a crime (9).

Despite the widely known and documented consequences of harmful alcohol use, the substance remains popular and widely accepted socially. This is due not only to cultural and moral factors but also to the biochemical and neurobiological mechanisms underlying the development of addiction. Among other things, alcohol affects the GABAergic system (by stimulating the main inhibitory neurotransmitter, producing a calming effect) and at the same time has an antagonistic effect on the glutamatergic system (10–12). Dopamine, serotonin and endogenous opioids also play an important role—their increase explains the formation of the reward effect and impaired urge control.

Abrupt cessation of alcohol use induces withdrawal symptoms—hyperactivity, tachycardia, increased blood pressure, muscle tremors, and even the risk of epileptic seizures (13). In addicts, the involvement of the ‘reward system’ mechanism is particularly important: the greatest dopamine release occurs already at the anticipation stage of consumption, explaining the increasing tension and drive to consume substances despite negative consequences.

Although the biological and psychological aspects of addiction are widely documented, in recent decades, increasing attention has been paid to the influence of social and environmental factors—including work—on the risk of harmful alcohol consumption.

The modern work environment, characterized by high demands, stress, pressure to perform, job insecurity, and frequent disorganized team relationships, is a significant area of exposure to factors that increase the risk of alcohol abuse. Alcohol is sometimes used as a readily available means of ‘coping’ with mental overload, isolation, or job burnout. In addition, in many industries and cultures, there is an acquiescence to drinking alcohol at company events or team-building events, which further increases the scale of the problem (14).

Therefore, an in-depth understanding of the biological, psychological, and social mechanisms that lead to harmful alcohol use in the work environment is important for the development of effective prevention strategies and the implementation of appropriate interventions at the level of entire organizations. A comprehensive approach, taking into account both individual and collective aspects, is one of the main public health challenges today.

Alcohol addiction in a work context has serious consequences for both the employee and the organization as a whole. It results in increased absenteeism, decreased productivity, more mistakes, and difficulties in maintaining good relationships with colleagues. In addition, the type of work performed, the conditions and hours of work, the form of employment, the level of workload, and occupational stress can all contribute significantly to an increased risk of alcohol misuse among employees (15–17).

The issue of alcohol consumption among employed people has long been an important topic of research, as work is not only a source of livelihood—people spend a large part of their lives in it, and its environment influences health behavior. In many cases, drinking is the result of a complex interaction of individual worker characteristics (e.g., psychological predispositions, stress coping skills) with characteristics of the work environment (18).

Predictors of increased risk of harmful alcohol consumption among employees have been widely described in the scientific literature. Young, childless, unmarried men with lower education and income are a particularly vulnerable group (19–21). Increasing attention is being paid to the role of environmental and organizational factors in the workplace, which can both contribute to and mitigate the risk of harmful alcohol use.

The target group for effective interventions in this area includes not only employees directly at risk of harmful alcohol use but also individuals in their professional environment—supervisors, coworkers, and specialists in human resources and occupational safety. Involving these groups in preventive efforts helps create a health-supportive work environment and enables early response to the symptoms of harmful alcohol use.

The aim of this review is to collect and systematize the current scientific evidence supporting the relationship between the working environment (working conditions and hours, form and stability of employment, level of remuneration, team atmosphere, and relationships) and employee alcohol consumption. To analyze the effectiveness of interventions implemented in the workplace to reduce harmful alcohol consumption.

## 2 Materials and methods

A literature review was conducted using the following scientific databases: ScienceDirect (Elsevier), MEDLINE (via PubMed), Scopus, and Google Scholar. The search was limited to articles published between 2020 and 2024. Search terms based on the Medical Subject Headings (MeSH) vocabulary were used as follows: alcohol consumption, workplace, risk factors, prevention, intervention, employees, and occupational health.

The phrase alcohol consumption was used as the primary keyword due to its umbrella function and its widespread adoption in the literature, which allows for capturing the full spectrum of alcohol use patterns and their consequences. More specific terms, such as harmful alcohol consumption or diagnostic labels (e.g., AUD), have a narrower scope and could limit the identification of relevant publications pertaining to the workplace context.

In research on broadly defined alcohol use, alcohol consumption constitutes a standard search term, enabling the inclusion of diverse definitions and theoretical perspectives. The addition of keywords related to the work environment further directed the search toward this specific domain.

The adopted strategy enabled the combination of a broad thematic scope with precise targeting of alcohol use in the workplace setting.

The literature review period (2020–2024) was intentionally chosen to capture the most up-to-date research on the prevention and intervention of harmful alcohol consumption in the workplace. During this period, numerous studies were published examining the effects of the COVID-19 pandemic on employee health, organizational changes, remote work, and new preventive strategies. Although valuable, older publications do not reflect current trends and the contemporary socio-occupational context. The remaining inclusion criteria were as follows: (1) peer-reviewed articles, (2) availability of the full-text, (3) publication in English, and (4) studies examining the relationship between the workplace and alcohol consumption or describing workplace-based interventions.

Studies were included if they examined the relationship between the work environment and alcohol consumption or described workplace interventions aimed at reducing harmful alcohol use among employees. Studies were excluded if they did not address these themes.

The initial search yielded 94 records. After screening titles, 53 publications were selected for further evaluation. Abstract screening resulted in the exclusion of 12 articles, leaving 41 articles for full-text review. After full-text screening, eight articles were further excluded for not meeting the inclusion criteria. Ultimately, 33 articles were included for detailed analysis: 20 focused on the association between workplace factors and alcohol consumption, and 13 described workplace interventions. Both review articles and original research articles were considered.

**Figure 1:**
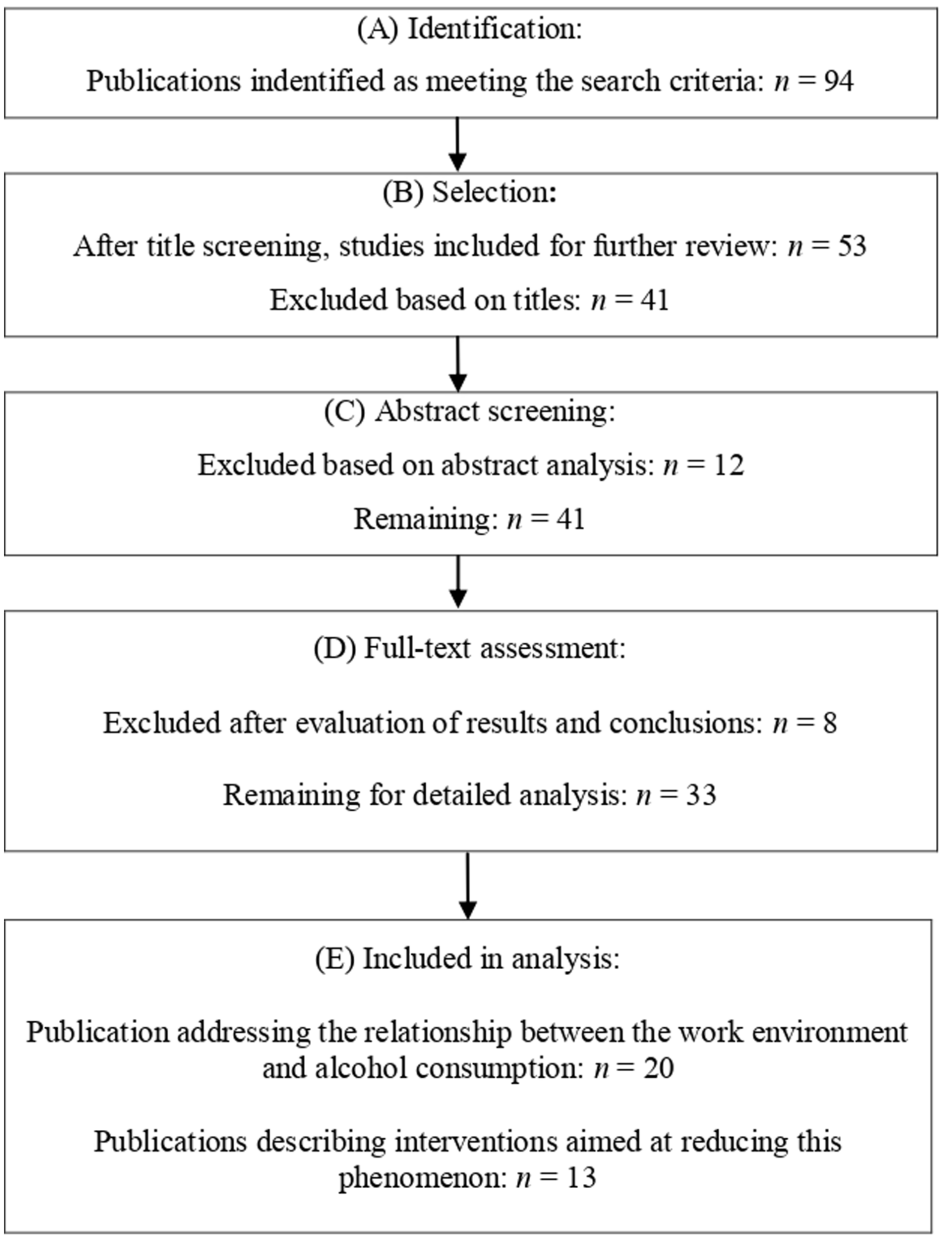
A flow diagram of the study selection process **(A)** identification, **(B)** selection, **(C)** abstract screening, **(D)** full-text assessment, and **(E)** included in analysis.

The following studies were excluded as follows: (1) those that did not meet the thematic criteria, that is, did not take into account the relationship between the work environment and alcohol consumption or did not describe workplace interventions, (2) non-peer-reviewed materials, (3) conference abstracts, and (4) articles lacking methodological information or data relevant to the review outcomes. The article selection process is presented in Figure 1. The literature review was conducted independently by two authors (M.G. and A.S.), and any discrepancies were resolved through joint analysis and agreement in the presence of the fourth author (B.K.).

Additionally, other relevant publications (e.g., foundational works or broader reviews outside the strict inclusion criteria) were cited in the discussion to provide context.

## 3 Results and analysis

Harmful patterns of alcohol use represent a serious and growing global health problem. As a result, increasing attention is being paid to protecting the mental health of workers. Table 1 presents a summary of studies conducted between 2020 and 2024 on workplace factors influencing employees’ alcohol consumption.

**Table 1:** Workplace factors influencing employees’ alcohol consumption.

No.	Name of study	Country	Characteristics of study participants	Description of study	Results
1.	Park S. et al. (2022) (21)	Korea	There were 2,058 participants (1,339 males and 719 females) aged 18–38 years: 18–29 years—966 individuals, 30–38 years—1,092 individuals. Employment status: permanently employed—1,579 people, temporary contract—479 people. All participants are young adult employees of companies employing up to 100 and more than 100 people.	Tools used were: CAGE (alcohol dependence risk assessment) and SRH (self-rated health). Binge drinking was defined as the consumption of at least seven drinks at a time by men or five by women, a minimum of two times a week.	The risk of alcohol abuse disorders increases with the duration of unstable employment, with the relationship being particularly strong among men.
2.	Thørrisen M. M. et al. (2022) (54)	Norway	A total of 5,388 participants (1,699 men and 3,689 women) aged 16–72 years participated. Employees of 22 companies were surveyed: private companies (*n* = 8), government companies (*n* = 6) and local government companies (*n* = 8).	The tools used were the AUDIT (early identification of alcohol problems) and the JCQ (measuring social and psychosocial aspects of work). Analyzed were (1) employment characteristics (hours, schedule, position, income), (2) support factors (demands, control, social support, competence of supervisors), (3) work travel (business trips, working from home or holiday home, commuting), and (4) social norms of drinking alcohol.	Approximately 10% of employees reported alcohol-related problems. Factors contributing to higher alcohol consumption included shorter working hours, being a supervisor (compared to a rank-and-file employee), full-time (vs. part-time) employment, higher income, higher levels of social support in the workplace, having a supervisor with less desirable leadership qualities, working from a holiday home and more liberal social norms of drinking alcohol in the workplace. The highest risk of alcohol problems in employees was observed with shorter working hours and liberal social norms in the work environment.
3.	Jonsson J. et al. (2021) (55)	Sweden	The study covered 2,743,764 Swedish residents aged 18–61 years.	Twenty-one employment trajectories (of which 10 were of low quality) were identified, based on the criteria of constancy, fluctuation and direction of mobility.	Low-quality employment trajectories were associated with increased risk of substance use disorders, including alcohol.
4.	El Haddad R. et al. (2022) (56)	France	Participants were 1,427 students who had never worked.	The AUDIT tool was used, and a 3-year follow-up with the question: “What is your current employment situation?.”	Risky alcohol consumption was associated with reduced employability, particularly hindering access to a first job.
5.	Baek S. U. et al. (2023) (57)	Korea	Participants were 9,611 workers aged 19–64 years.	Tools used: AUDIT, job satisfaction survey (5-point Likert scale) and assessment of type of employment (permanent, temporary, casual).	Temporary employment and job dissatisfaction were associated with an increased risk of alcohol use disorders.
6.	Murray K. et al. (2021) (58)	Ireland	Participants included 717 GAA (Gaelic football) players, mean age 24 years.	The AUDIT tool and a self-administered questionnaire on sociodemographic data and alcohol behavior were used.	Of those surveyed, 96% consumed alcohol. Players consumed larger amounts of alcohol in the off-season; 93% reported episodes of binge drinking and 65% experienced alcohol-related harm. Excessive alcohol consumption, alcohol-related harm and binge drinking are common among elite athletes, especially in the off-season.
7.	Mou N. L. et al. (2023) (59)	China	Participants were 1,375 Filipina women aged 18–65 years, living and working in Macau (China).	The AUDIT tool, the DSM-5 self-assessment scale 20 of PTSD symptoms (PCL-5) and the DSM-5 Gambling Disorder Measurement Scale were used.	Migrant workers are susceptible to PTSD symptoms and at risk of alcohol and gambling addiction both before, during and after migration. Major stressors include exploitation, abuse, precarious employment conditions, prolonged separation from family and difficulties in securing family livelihoods.
8.	Thern E. et al. (2024) (60)	Sweden	Participants were 339,403 people aged 19–30 years with tertiary education (3 years after graduation), living and working in Sweden.	Data from the Swedish Insurance and Labour Market Register and the National Hospital Register were used, using the Swedish versions of ICD-9 (1987–1996) and ICD-10 (from 1997). The diagnoses included were alcohol-related mental and behavioral disorders (ICD-9: 291, 303; ICD-10: F10), alcoholic liver disease (ICD-9: 571; ICD-10: K70) and alcohol toxicity (ICD-9: 980; ICD-10: T51).	Precarious employment in young adulthood is associated with an increased risk of alcohol-related morbidity later in life.
9.	Grandey A. A. et al. (2020) (61)	USA	Participants were 1,592 service workers aged 18–65 years employed in the USA. Participants were divided into those with limited personal contact with customers and frontline workers.	A telephone interview was used. The following were examined: (1) self-control based on an impulsivity scale and (2) frequency of alcohol consumption in the past 12 months within 2 h of leaving work.	Employees who have daily contact with customers and provide a ‘service with a smile’ need to display greater emotional self-control and thus have a greater tendency to consume alcohol harmfully. People with high levels of impulsivity are particularly prone to harmful drinking.
10.	Azagba S. et al. (2021) (62)	USA	A population of American adults was included, using data from the Behavioral Risk Factor Surveillance System (BRFSS) from 2001 to 2017.	The analysis was based on a publicly available database.	The findings do not support the assumption that having health insurance contributes to more problematic alcohol use. Existing data indicate that health insurance is not significantly associated with levels of alcohol consumption.
11.	Kim J. Y. et al. (2021) (63)	Korea	Participants were 815 junior military personnel with up to 5 years of service, aged 18–24 years.	An offline survey was used, which included a tactical conflict scale and the Alcohol Use Disorders Identification Test (AUDIT).	Experiences of victimization in the workplace can lead to feelings of anger, both of which are considered predictive of alcohol misuse.
12.	Hsu S-T. et al. (2022) (64)	Taiwan	Participants were 90 individuals recruited from the outpatient substance abuse treatment unit at Kaohsiung Municipal Psychiatric Hospital.	The Workplace Substance Reuse Questionnaire (WSRQ) alcohol version (WSRQ-Alc) was used, including factors: 1. Work environment, 2. Workload, 3. Social interactions in the workplace, 4. Other drinking signals.	Recurrent alcohol use in the workplace correlates with job instability, manifested by frequent job changes and part-time employment, among other factors. Patients characterized by a longer duration of alcohol use and employment instability have been shown to be significantly more at risk of reusing alcohol in the workplace.
13.	Oesterle S. et al. (2023) (65)	USA, Australia	Participants included 751 young adults from the USA and 777 young adults from Australia.	A self-report survey was used, collected using an adapted and expanded version of the Communities that Care Youth Survey questionnaire.	Young adults in Australia were almost two times as likely to drink at work during working hours as in the USA, almost three times as likely to drink after working hours, and 25% more likely to come to work hungover. The percentage of those working under the influence of alcohol was similar in both countries. The availability of alcohol in the workplace and the lack of a written alcohol policy, especially a total ban on drinking, increased the likelihood of alcohol consumption by three times. Perceived tolerance or encouragement of drinking in the workplace increased the risk of drinking at work by almost five times.
14.	Bonsaksen T. et al. (2021) (66)	Norway	Participants were 6,620 employees aged 18–71 years, including: 143 top management, 1,161 middle management and 5,316 permanent employees.	The instruments used were: 1. The Job Content Questionnaire (JCQ) to measure psychosocial work environment, 2. A 16-item shortened version of the Effort-Reward Imbalance Questionnaire, 3. The AUDIT screening tool, 4. One question from the Work Performance and Activity Impairment Questionnaire (WPAI): “How has your alcohol consumption affected your productivity at work in the past 7 days?”	Employee over-engagement was associated with an increased risk of alcohol-related presenteeism in the past week.
15.	Cannizzaro E. et al. (2023) (67)	Italy	640 participants aged 25–65 years, including 321 men and 319 women, with a minimum of 4 years’ experience of working remotely, participated.	The AUDIT screening tool was used for early identification of alcohol problems.	The imposition of remote working during the pandemic negatively affected the psycho-physical wellbeing of employees, causing stress that promoted risky behavior. In the study population, the COVID-19 pandemic affected alcohol consumption patterns and frequency, with increased consumption increasing health risks.
16.	McKetta S. et al. (2023) (68)	USA	The study involved 16,571 women aged 19–45 years with high employment status.	The questions used: 1. A drinking frequency question: “How many times have you had any alcoholic beverage—more than a few sips—in the past 30 days?” 2. A drinking question: “Think about the last 2 weeks. How many times have you had five or more drinks in a row?” 3. Occupational status was based on classifications used by the Bureau of Labor Statistics, corresponding to the category “Managerial, professional, and related occupations.” 4 Structural sexism was operationalized using a measure based on factor analysis, consisting of indicators of gender inequality.	Work was associated with more frequent drinking and a higher risk of binge drinking, especially in environments with low levels of sexism. At the highest levels of sexism, no differences in alcohol consumption were observed by occupational characteristics. High-status occupations were associated with a higher frequency of drinking in low-sexism environments. The gender composition of the occupation was not significantly related to alcohol consumption, regardless of the level of structural sexism.
17.	Lee M. et al. (2021) (69)	Korea	Participants were 13,858 workers (men and women) aged 20–65 years who underwent workplace mental health screening at the Institute of Mental Health at Kangbuk Samsung Hospital between 2014 and 2019. The respondents were employed as office workers in one of 52 public institutions or as employees of large manufacturing companies in the country.	The instruments used were: 1. AUDIT, 2. Suicidal thoughts assessment based on one of the items on suicidal thoughts and attempts included in the Korean National Health and Nutrition Survey, 3. Korean version of the Center for Epidemiological Studies Depression Scale (CES-D), 4. Korean version of the Beck Anxiety Inventory (BAI), 5. WHO Quality of Life Tool (WHO-QOL), 6. Daily Life Stressors Scale (DLSS), 7. Korean Occupational Stress Scale (KOSS), 8. Korean version of the Connor-Davidson Resilience Scale (K-CD-RISC).	The results showed that alcohol dependence and related problems were associated with depression and suicidal thoughts. Identifying and treating addiction may help to reduce psychological problems, especially suicidal thoughts in employees, and reduce social costs.
18.	Thern E. et al. (2024) (70)	Sweden	Participants were 54,378 people who took part in Feelgood’s (a Swedish occupational health services company) annual survey on health, work environment and lifestyle (HALU) habits between 2012 and 2023.	The surveys used were: 1. AUDIT, 2. Workplace information, which was used by Feelgood to define the industry according to the Swedish Standard Industrial Classification (SNI).	Differences in hazardous alcohol consumption between industries were noted. The highest prevalence was in the industries of accommodation, catering, arts, entertainment, recreation, construction, finance, and insurance. Compared to education, most industries had an increased risk of hazardous alcohol consumption.
19.	Rospenda K. M. et al. (2023) (71)	USA	Participants were 4,832 university employees, including 2,416 men and 2,416 women, with a mean age of 40 years. The study was conducted between 1996 and 2007 and then again between 2020 and 2021 with a sample of 2,352 employees.	The instruments used were: 1. Modified version of the Sexual Experiences Questionnaire (SEQ) to measure sexual harassment at work, 2. 29-item Generalized Workplace Harassment Questionnaire (GWHQ), 3. Stress index calculated by summing the number of different stressors experienced in the 12 months preceding the survey, 4. Assessment of alcohol abuse based on indicators: frequency of drinking to intoxication in the past 12 months or consumption of six or more drinks.	Approximately one-third of the respondents experienced generalized harassment (GH) or sexual harassment (SH) at work. Chronic exposure to SH and GH has significant long-term effects on psychological distress and alcohol abuse. It has also been shown that previous exposure to SH and GH is associated with current heavier drinking and has a stronger effect than current stressors.
20.	Baek S. U. et al. (2024) (72)	Korea	Participants were 10,206 young people aged 15–29 years between 2016 and 2020.	The items used were: 1. CAGE questionnaire to assess problematic alcohol use, 2. Weekly schedules of working hours.	Of the 27,646 participants, 5% worked 35 h/week, 51% worked 35–40 h/week, 24% worked 41–48 h/week, 11% worked 49–54 h/week, and 9% worked 55 h/week. Working more than 40 h/week increased the risk of alcohol dependence among young Koreans, especially during the year after starting to work at this rate.

Source: Authors’ own study based on Refs. (21, 54–72).

Analysis of the studies presented in Table 1 indicates that harmful alcohol use among employees is particularly influenced by:

- Unstable and temporary employment—long-term work on temporary contracts or casual jobs increases the risk of developing alcohol problems, especially in young adults.
- Job dissatisfaction and negative atmosphere—lack of social support, poor quality of relationships with superiors or bullying significantly correlate with more frequent and more harmful drinking.
- Long or unusual working hours—overtime, shift work, or remote working are associated with higher alcohol consumption, as are excessive workloads and perceived stress.
- Liberal employer policies toward alcohol consumption—the lack of a clearly defined and enforced alcohol policy, as well as easy access to alcohol in the workplace, significantly increase the risk of excessive consumption.
- Occupational position and socio-demographic aspects—people with lower qualifications, lower wages, young men, single people, and workers in specific industries, such as catering, construction, or finance, are at increased risk.
- Additional factors—chronic stress, experience of violence or harassment at work, experience of post-traumatic stress disorder (PTSD), and significant random events (e.g., COVID-19 pandemic).

The aforementioned studies also emphasized the role of awareness of one’s own problems and the limitations of therapy and prevention in the workplace. Identifying risk factors makes it possible to target effective preventive measures and promote a healthy working environment.

The work environment, by eliminating the factors mentioned and implementing clear standards and preventive measures, can effectively reduce harmful drinking and its consequences—both individual and organizational.

Factors contributing to hazardous alcohol consumption by employees can be divided into those related directly to the conditions of employment (such as work schedule and hours, position, workload, or salary) and those related to job demands and the working environment (such as stress levels, job control and appraisal, management style, business travel, remote working, interpersonal relationships, and prevailing social norms—including tolerance toward alcohol consumption). Awareness of these factors makes it possible to more effectively eliminate those that increase the risk of alcohol misuse and to create clear policies and prevention programs, so that the workplace can become an environment that supports employee health.

The vast majority of therapeutic interventions related to the treatment of alcohol dependence take place within the healthcare system, as medical procedures. In practice, however, many excessive drinkers do not recognize their problem and do not seek help. This is due to the psychological mechanisms of addiction, such as:

- The mechanism of compulsive emotion regulation—the desire to drink arises in response to difficult emotions, the need for quick relief, the desire for repeated pleasure, the relief of stress or negative emotional states, and the expectation of positive outcomes and ‘dissociation’ from problems (22).
- Illusion and denial mechanism—the addict does not perceive or denies the harmful effects of their drinking, rationalizes and minimizes symptoms, convincing themselves and those around them that there is no problem and treatment is unnecessary (23, 24).
- The mechanism of a diffuse and split self—in the state of inebriation, there is a sense of control and “power” (the “Powerful Self”), during sobriety, there is suffering, shame, and a sense of inferiority (the “Fallen Self”). In effect, the person balances between these states, replicating the cycle of escape into alcohol (24–26).

It is estimated that as many as one in ten workers need intervention or support related to problematic alcohol use (16, 27), due to impaired health, absenteeism, reduced productivity (presenteeism), and higher risk of accidents and injuries (26–28). As much of adult life is spent at work, the workplace is an important setting for early detection and effective interventions for those at risk of developing harmful alcohol habits (16, 27, 29).^3^

Using this focus on alcohol prevention, Table 2 outlines the types of interventions that can be undertaken in the workplace.

**Table 2:** Alcohol dependence prevention interventions that can be undertaken in the workplace.

No.	Name of study	Country	Characteristics of study participants	Description of the survey	Results
1.	Sunami T. et al. (2022) (29)	Japan	Participants were 100 employees from six companies operating in four sectors (electrical engineering, transportation, construction, and steel industry), allocated to an intervention or control group (50 participants each). Inclusion criteria included age ≥ 20 years, regular Internet access, ability to use e-mail and a score of ≥8 on the AUDIT test.	A SNAPPY-DOC program for self-monitoring of alcohol consumption was developed. Participants in the intervention group recorded their daily alcohol consumption in it for 4 weeks, and received a weekly e-mail with feedback and educational information about alcohol. Everyone in both the intervention and control group logged into SNAPPY-DOC at the beginning of the study and reported their alcohol consumption over the previous 7 days using the TLFB method. The control group additionally listened to a lecture on the harms of alcohol, but did not continue to use the program for daily monitoring for a further 4 weeks.	There was a significantly greater increase in the number of alcohol-free days in the intervention group and a decrease in total weekly alcohol consumption and AUDIT score compared to the control group. The SNAPPY-DOC program, therefore, reduced weekly alcohol consumption mainly by increasing the number of days of abstinence rather than by reducing the number of drinks consumed on drinking days.
2.	Thørrisen M. M. et al. (2021) (73)	Norway	Participants were 779 risky drinkers from 22 Norwegian companies (age 16–72 years, actively employed, speaking Norwegian). The mean age was 40.3 years; 48.9% were male, 51.1% female. The majority had a university education (72.1%). Alcohol consumption and its consequences were assessed using the AUDIT test.	Participants were invited to a medical examination, after which they were randomly assigned to one of three groups: (1) a brief intervention with motivational interviewing and an alcohol brochure; (2) an e-health program on a digital platform and an alcohol brochure; or (3) a control group receiving only the brochure. The motivational interview intervention included two interviews, the digital intervention included 62 online sessions over 6 months, and the booklet provided basic information about the effects of alcohol on the body.	In the study, four out of 10 employees who drank at risk were willing to participate in preventive interventions in the workplace. These individuals did not differ significantly from those unwilling to participate. Employees, compared to managers, showed less willingness to participate, highlighting the need to ensure anonymity and safety and to destigmatize alcoholism prevention interventions.
3.	Berge L. I. et al. (2023) (74)	Sweden	The EWA RCT involved 200 women with alcohol use disorders; mean age was 42.5 years.	The effect of an early treatment program for women with alcohol dependence (EWA) on sick leave, income, unemployment and early retirement up to 25 years after starting treatment was analyzed.	The 25-year study found that a women-only alcohol treatment program (EWA) had long-term benefits in terms of sick leave and income. Compared to the treatment in a mixed environment (TAU) group, women in the EWA group had a slower increase in sick leave up to 21 years after treatment and higher income in the first 8 years, especially younger participants. The results indicate that intensive, dedicated therapy for women can significantly improve long-term occupational functioning.
4.	Blake H. et al. (2023) (31)	International study	Participants included 362 respondents aged 21–65 years, 87.8% of whom worked shifts. Professional groups included doctors in training (48.6%), nurses (22.4%), and physicians (18.5%).	This was an open, cross-sectional, international survey using a web-based questionnaire (closed and open questions). Participants were emergency care workers (UEC) over the age of 18, representing a variety of professions and coming from any country or region.	Service workers are open to alcohol prevention activities, but implementation in the UEC setting has faced difficulties due to workload and staff shortages. They call for clear guidelines, better staff support or dedicated health promotion teams, SBIRT training, appropriate interview settings and more effective referrals to specialized services. Successful SBIRT implementation requires a sustained commitment of time and resources to prevention across the care system.
5.	Elling D. L. et al. (2023) (49)	Sweden	Participants included 853 people from 11 workplaces (393 women, 460 men). They were divided into an intervention group (*n* = 267; mostly men, higher education, higher mean AUDIT score) and a control group (*n* = 586; mostly men, secondary education, lower mean AUDIT score). Observation lasted 12 months.	The alcohol prevention program ‘Alcohol Policy and Managers’ skills Training’ (APMaT) comprised two phases: first, HR staff and managers from each organization refined an alcohol policy and developed a plan for its implementation, then managers attended a two-part workshop—the first part focused on knowledge about alcohol in the workplace and its effects, and the second on developing skills to recognize the early signs of risky drinking and how to have difficult conversations with employees.	A study of the APMaT program showed a greater decrease in hazardous drinking in the intervention group, although the difference was not statistically significant. The program also increased managers’ willingness to intervene, which may improve alcohol policy implementation and employee awareness. Future prevention programs should involve employees at all levels and take into account norms and organizational culture at the design stage.
6.	Elling D. L. et al. (2022) (75)	Sweden	187 managers participated—a control group (*n* = 70, mainly tertiary education) and an intervention group (*n* = 117, mainly primary education). Willingness to early alcohol intervention was assessed using three questions on a 5-point Likert scale. Differences between groups at baseline and after 12 months were analyzed. Both groups reported high or very high confidence in undertaking early intervention and the need to confirm incidents of hazardous drinking by employees.	A workplace intervention was implemented by introducing an Alcohol Policy and Managers’ skills Training (APMaT) in recognizing and responding to early signs of hazardous drinking to prevent and reduce alcohol-related harm.	The APMaT program is effective in increasing the propensity of managers to intervene early against hazardous alcohol use at work. There was a significant increase in the intervention group, including a 50% greater confidence of managers to initiate such action compared to the control group.
7.	Manning W. et al. (2021) (76)	Australia	Participants included 1,309 people aged 18–75 years (758 females, 538 males, 13 people with a different gender identity). Recruitment was conducted through Facebook advertising, directing to the screening questionnaire. Inclusion criteria were age ≥18 years, AUDIT score ≥8, ownership of an Android or iOS smartphone with an Australian phone number, and a desire to reduce or stop drinking.	When the app was first launched, participants provided their alcohol consumption data for the past month and week, selected alcohol-related and motivational images, and then began their first ApBM session. Before and after each session, they measured their level of alcohol craving on a VAS scale; with a score of 90 or higher, an addiction helpline contact was displayed. Participants completed at least two ApBM sessions per week for 4 weeks, reporting weekly the amount of alcohol consumed. At the end of the program, they completed a questionnaire (CEQ-F, SDS, uMARS) and 1 month later a final questionnaire on their alcohol consumption in the last month and week.	The average number of days of alcohol consumption gradually decreased over the 4 weeks of SWiPE use. The program showed high acceptability and encouraging effects—a reduction in the number of days and amount of alcohol consumed, as well as a reduction in rates of dependence and alcohol craving. These results suggest the potential of SWiPE as an effective public health tool for risky drinkers, warranting an RCT.
8.	Lubman D. I. et al. (2022) (77)	Australia	Participants included 344 people aged 18–73 years (167 women, 177 men). The intervention group comprised 173 participants (50.3%) and the active control group 171 (49.7%). Individuals with problematic alcohol use (AUDIT > 6 for women, > 7 for men) were recruited from across Australia via social media.	Participants were randomly allocated to an intervention group or an active control group. The intervention group completed four to six weekly, 30-50-min telephone sessions with the same counsellor, including emotion regulation, anger management, urges, sleep hygiene, mindfulness and support for anxiety and lowered mood. The control group received guidance on alcohol consumption, brochures on coping with stress and four short follow-up calls (approximately 5 min) with information on available forms of support.	The results of the study indicate that the telephone intervention is beneficial in reducing hazardous drinking, problem severity, risky drinking patterns and total consumption, especially when it includes at least two sessions. Although there was no significant difference in the main outcome between the groups after 3 months, the telephone intervention was found to be effective in infrequent treatment seekers. This means that this easily scalable telehealth model can effectively help to reduce the treatment gap for problem drinking.
9.	Koffarnus M. N. et al. (2022) (32)	USA	Participants included 92 people of both sexes, predominantly male, from of whom 56 met inclusion criteria (age over 18 years). Participants met DSM-5 criteria for alcohol use disorders, did not meet DSM criteria for other substance use disorders (except caffeine, marijuana, and nicotine), scored below 23 on the Alcohol Withdrawal Symptoms Checklist, were willing to pay a US$75 deposit into an incentive fund, and expressed a desire to reduce or stop drinking.	Participants were recruited through advertising in public spaces and on websites. The randomized, parallel study included two phases: a 7-day monitoring phase, in which participants reported their alcohol consumption and withdrawal symptoms daily via text message, and a 21-day treatment phase, during which participants took breathalyzer measurements three times a day: upon waking, before bedtime and once during the day (at least 6 h apart, between 5 a.m. and midnight). Sample collection reminders were sent via SMS. Additional breathalyzer checks took place before and after the follow-up phase, and at 1, 2, 3, and 6 months after the end of treatment. In the baseline group, participants paid a US$75 deposit and received a US$1 daily reward for regular sample submission. In the control group, abstinence was incentivized through a bonus system—US$5 was received for the first day of abstinence, the amount increased by US$1 with each subsequent day, and the total possible amount over the study period was US$350.	The study found that daily, immediate financial rewards for remotely confirmed abstinence (breathalyzer samples at zero), significantly increased the number of days of abstinence (86%) compared to rewards for sample delivery alone (44%), with an odds ratio of 8.2. This confirms the effectiveness of such an incentive intervention. However, the requirement for a deposit may exclude the most intensive drinkers or those in poverty, so this condition may limit access to this method for precisely those most in need. Despite the high potential of remote monitoring, these models should be tailored to not exclude those with the greatest financial and health barriers.
10.	Barticevic N. et al. (2020) (78)	Chile	182 people over 18 years of age, of mixed gender, seeking treatment participated. Participants met DSM-5 criteria for alcohol use disorders and criteria for harmful alcohol use: at least five instances of heavy drinking in the past month (≥5 drinks at a time for men, ≥4 for women) or an average consumption of at least 14 drinks per week for men and seven for women.	The study was designed to assess the effectiveness of short-term motivational treatment (BMT) compared to enhanced standard care. Participants in the intervention group received a computerized version of motivational enhancement therapy, comprising four 45-min sessions led by a psychologist with at least 3 years’ experience in primary care. The first three sessions, delivered over a 6-week period, focused on actively supporting behavior change, while the final session served to close and summarize the therapeutic process. If necessary, participants could benefit from up to two additional sessions before the end of therapy. The control group consisted of people receiving standard addiction therapy.	It was assumed that Behavioral Management Therapy (BMT) would result in at least a 26% reduction in alcohol consumption compared to standard therapy. BMT was developed as a psychosocial intervention based on the best available evidence of effectiveness. Participants in BMT therapy are also provided with additional recovery support services, such as social support or medical consultations, to comprehensively support the treatment process.
11.	Kelly J. F. et al. (2020) (79)	Review of studies.	Twenty-seven relevant studies involving a total of 10,565 participants were identified (21 randomized / quasi-randomized studies, 5 non-randomized studies and one economic study). The mean age of participants in each study ranged from 34.2 to 51.0 years.	Twelve-step facilitation (AA/TSF) interventions were compared with treatments based on other theories, such as cognitive-behavioral therapy. The aim was to examine differences in effectiveness between the 12-step program-based approach (AA/TSF) and alternative therapeutic approaches, particularly cognitive-behavioral therapy.	A comparison of AA/TSF (Alcoholics Anonymous/Twelve-Step Facilitation) with other clinical interventions showed that AA/TSF results in higher rates of continuous abstinence at 12, 24, and 36 months. In addition, AA/TSF programs can result in significant savings in healthcare costs while effectively supporting the maintenance of alcohol abstinence.
12.	Garnett C. et al. (2021) (80)	United Kingdom	Participants were over 18 years of age, 55% of whom were female, living in the UK and interested in reducing or stopping drinking alcohol.	The Drink Less app, designed to reduce alcohol consumption, gained widespread national media attention in the UK behind the involvement of a well-known 51-year-old TV and radio presenter. In the 66 weeks prior to the media exposure, the app was downloaded by 8,617 users and in the subsequent 23 weeks following the media campaign, it was downloaded by a staggering 18,959 people. A total of 27,576 users of the Drink Less app were reached in the ongoing research.	The publicity of the Drink Less app by celebrities and national media in the UK translated into an increase in downloads, logins and greater user engagement, particularly among men in mature adulthood. At the same time, there was a significant decrease in average AUDIT scores among users, suggesting a positive promotional impact on the app’s effectiveness. This indicates that leveraging celebrity and media influence can be a very effective strategy for popularising digital health interventions.
13.	Traxler H. K. et al. (2022) (81)		Participants were 106 Amazon Mechanical Turk employees, mainly male (74.5%), Caucasian (81.1%), with a mean age of 33.49 years. The majority of participants reported consuming 3–4 alcoholic beverages per day, and approximately 36.8% drank this amount daily. All expressed a desire to reduce or stop drinking.	Contingency management (CM), a method of offering financial incentives once abstinence from alcohol, cigarettes and/or illicit substances has been confirmed, is among the most effective and empirically best documented approaches in the treatment of substance use disorders. However, the main barrier to implementing this method is the financial cost associated with its implementation. In the study, participants were asked to indicate how likely they were to reduce or stop drinking alcohol if they received a monetary reward for abstinence for 1 day, 1 week, 1 month, or 1 year.	Participants were more likely to report maintaining abstinence with higher financial incentives and shorter required periods of abstinence. They were also more likely to buy access to treatment when it was more effective and/or cheaper. With the increasing availability of contingency management (CM), purchasing tasks can support professionals in determining optimal treatment conditions and costs based on data.

Source: Authors’ own elaboration based on Refs. (29, 31, 32, 49, 73–81).

A review of international research indicates that effective prevention of alcohol dependence in the work environment can take very different forms—from individual digital tools, to specialized training, to extensive programs involving entire organizations. The key findings are as follows:

- Modern technology helps—mobile apps and online self-monitoring programs (e.g., alcohol logging or online workshops) can make a real difference in reducing drinking, especially when they provide individual support, progress monitoring, and regular reminders.
- Importance of employer and supervisor support—the effectiveness of programs increases when managers and HR are involved in initiatives, and alcohol policies are clearly articulated and enforced. Training for managers and workshops on recognizing risky alcoholic behavior increases confidence to engage in difficult conversations and early interventions.
- Accessibility and anonymity increase effectiveness—employees are more likely to participate in programs if they feel anonymous and confident that their data is safe. Destigmatizing the subject promotes effective interventions.
- Individualization and tailoring—programs profiled by gender, age, position, or severity of the problem are more effective than one-size-fits-all solutions.
- Use of short, motivational interventions—a short conversation with a member of staff, psychological support or even a few telephone sessions can make a significant difference in changing alcohol habits, especially in at-risk groups or those who rarely reach out for professional support.
- Economic and motivational incentives—additional financial incentives (abstinence bonuses, rewards for adhering to the program) are effective in motivating people to reduce their alcohol consumption, although they have their limitations—especially in lower-income populations.
- Effect of interventions seen at work and beyond—research shows that effectively implemented, long-term interventions not only reduce alcohol consumption but also improve wellbeing, increase productivity, and reduce dismissals and accidents at work.

The variety of approaches and tools that can be introduced in the workplace enables effective prevention and support for people with risky or harmful alcohol habits. Multi-level programs, combining educational activities, technical support, engaging leaders, and individualized approaches to meet the needs of employees, are most effective. The scientific literature is still lacking many detailed studies on the implementation of alcohol prevention programs in the workplace (30). However, a review of the studies presented in Table 2 shows that the interventions undertaken range from state-of-the-art, web-based tools, to a combination of electronic sessions with individual medical care, therapy or psycho-educational lectures, to financial incentives for employees maintaining abstinence (31, 32). Most of these programs have been shown to be effective in reducing risky drinking among employees and make a valuable contribution to health promotion in the work environment.

The most important limitations of the interventions analyzed are mainly the short follow-up time and the lack of data on the maintenance of effects in the long term. This means that it is often unclear whether the beneficial results have a lasting effect or whether they fade after the program ends. An additional challenge remains the recruitment of participants—especially those who consume alcohol in a risky manner and are afraid of stigmatization or are not ready to take advantage of the available help (33, 34). In this context, anonymous, web-based prevention programs gain particular advantage, as they minimize fear of peer assessment, break down barriers related to the availability of support and can more effectively reach groups most at risk of harmful alcohol use.

Despite their documented effectiveness, the implementation of prevention programs in the work environment often faces numerous difficulties. Establishing clear guidelines is crucial here—from the introduction of a coherent alcohol policy, to education and health promotion activities (including rapid Internet interventions), to tailoring solutions to the specific characteristics of the industry and organization, especially in sectors such as hospitality, catering or others where access to alcohol is easier (35). However, the ultimate success of these measures depends largely on the level of commitment of managers, who should be actively involved in the development and long-term maintenance of prevention programs. It is worthwhile to draw on good practice in this context, such as the Swedish experience in prevention and health policy (36).

### 3.1 Practical implications

Analysis of successful alcohol prevention programs shows that it is possible to develop a universal model that any employer can implement once it has been appropriately adapted to the specifics of their organization. Such a program is worth planning as a basic solution, offering in addition optional elements that can be introduced as needs and opportunities—both organizational and financial—arise.

It is crucial that the implementation of the program goes hand in hand with the analysis and elimination of risk factors for harmful alcohol consumption in the workplace. In practice, the starting point should be the development and adoption of an alcohol policy at all levels of the organization and its integration into day-to-day operational activities.

At the operational level of management, it is necessary to define a timetable for implementation, to designate those responsible and to select the scope of the program—from a basic to an expanded version. Continuous monitoring of the implementation of the policy, ongoing evaluation of its effectiveness, and regular, preferably annual, evaluation to make necessary improvements are also essential.

### 3.2 Suggested checklist for implementing an alcohol prevention program in the workplace

1. Diagnose needs and risks—identify specific risk factors in the organization (e.g., stress, availability of alcohol, working hours). Conduct an anonymous survey or consultation with employees.
2. Develop an alcohol policy—prepare a clear, written alcohol policy that applies at all levels of the organization. Establish rules for dealing with violations.
3. Select a team responsible for implementation—designate persons responsible for coordinating the implementation of the program (e.g., HR, management, trade union representatives).
4. Define the scope and timing of implementation—set a timeframe for the program and the stages of implementation (e.g., pilot, full implementation, and annual evaluation). Decide whether you are implementing a basic program or an extended program with additional elements.
5. Tailor the program/procedures to the specifics of the organization—tailor solutions to organizational and financial capacity. Take into account job specifics and potential obstacles.
6. Education and communication—conduct training and workshops for staff and management. Provide access to information material (e.g., brochures, posters).
7. Implement preventive actions—launch prevention activities, for example, self-monitoring apps, counseling, consultations, and the possibility of external support programs. Establish procedures for rapid intervention when problems are identified.
8. Ensure anonymity and data protection—ensure confidentiality of program participation and protection of personal data.
9. Monitor and evaluate program implementation—identify and implement indicators to measure effectiveness (e.g., number of trainings, notifications, and cases of early problem detection). Collect employee feedback on the program.
10. Regular evaluation and improvement—conduct an annual evaluation of the program’s effects. Make adjustments and improvements based on results and feedback.

## 4 Discussion

Introducing an effective alcohol policy in the workplace is one of the key challenges of health prevention at the organizational level. It is clear from the literature and analyses of international experiences that the success of such measures depends primarily on the commitment of managers and their real belief in the values and benefits of implementing these solutions (37). It is also of utmost importance to systematically prepare and develop the competencies of managers, who play a central role in both the initiation and long-term maintenance of effective health policies in the workplace.

### 4.1 The role of addressing psychosocial risks

Tackling psychosocial risks—such as chronic stress, interpersonal conflicts, or deficits in social support—not only reduces the risk of developing addictions (including alcohol) but also significantly reduces the incidence of mental disorders, musculoskeletal diseases, traumatic incidents, cardiovascular conditions, and even the risk of premature mortality (38). Thus, measures aimed at improving psychosocial wellbeing are an effective investment not only in the health of the employee but also in the overall potential and productivity of the entire organization.

### 4.2 Lessons from international experience

It is worth noting that the value of preventive interventions has been recognized and implemented in the policies of a number of countries, such as the Scandinavian countries, the UK, Canada, Japan, Colombia, and South Korea, where psychosocial risk management is an integral part of health and safety regulation (39). In the United States, the Healthy Work campaign has set new standards by identifying psychosocial risks and widely promoting awareness of the impact of occupational stress on workers’ health (40). Experiences such as these show that systemic action and widespread education yield tangible results.^4^

### 4.3 Effectiveness of workplace interventions

In the literature, the concept of effective interventions for preventing alcohol-related problems in the workplace is defined in diverse ways, which makes it difficult to operationalize the term unambiguously. As noted in the systematic review by Webb et al., the effectiveness of interventions depends not only on the type of program (e.g., training, screening, and brief interventions) but also on how well it is embedded within a broader organizational policy that includes elements of employee support, confidentiality, and collaboration with management. The authors emphasize that multicomponent programs—combining educational, monitoring, and structural measures—are more effective than single, information-based initiatives (41).

In the Cochrane review by Liira et al., attention was drawn to the fact that interventions aimed at preventing job loss and reducing the negative health consequences among individuals with alcohol misuse are most effective when tailored to the occupational context and linked to employee health policies. In this perspective, effectiveness refers not only to a reduction in alcohol consumption but also to improvements in employment indicators, psychosocial functioning, and workplace relationships (42). In light of these findings, concepts such as alcohol policy, effective preventive strategies, and multicomponent programs should be understood relationally—as components of an integrated approach that encompasses both individual-level and structural interventions.

Analysis of recent research clearly indicates that interventions targeting the work environment have multidimensional benefits—both from the perspective of the individual employee, the employer or the organization as a whole (28, 43). Comprehensive interventions that include a clear alcohol policy, educational programs, specialist counseling and regular structured health checks form the foundation of a healthy and safe working environment (44). It is worth noting that even relatively simple solutions, such as regular sobriety checks or access to anonymous support and prevention programs, make it possible to significantly reduce alcohol levels among employees (45). The results of the meta-analysis conducted by Fellbaum et al. confirm that environmental interventions aimed at reducing alcohol consumption in the workplace are significantly effective, especially when they combine educational components with behavior-change support activities and elements of participant progress monitoring and evaluation (46). Authors emphasize that what is crucial is not only the scope of the interventions but also their long-term nature and their alignment with the organizational characteristics of a given institution. In turn, studies published in *International Journal of Workplace Health Management* indicate that programs based on employee participation and managerial involvement yield more sustainable outcomes in reducing alcohol consumption and improving employees’ psychological wellbeing (47). Taken together, these findings suggest that effective alcohol prevention in the workplace requires a systematic approach that integrates education, psychological support, and control measures. Such solutions not only help maintain a high level of safety but also strengthen an organizational culture based on responsibility and mutual trust.

These findings are supported by recent research: a systematic review by Morse et al. demonstrated that universal and selective interventions—such as health promotion programs, brief interventions, and large-scale screening initiatives—can significantly reduce alcohol consumption among employees (48). Moreover, results from randomized trial by Elling et al. indicate that multicomponent programs incorporating organizational policies and managerial training may facilitate early identification of risks and reduce alcohol use, even if the initial individual-level effects are moderate (49).

Based on this, it can be concluded that the literature—spanning both classic and more recent studies—clearly indicates that workplace-focused interventions can yield multidimensional benefits. Comprehensive approaches that combine organizational policies, education, monitoring, and intervention strategies form the foundation of a healthy and safe work environment, thereby supporting reductions in alcohol consumption, improving occupational functioning, and mitigating alcohol-related harms.

### 4.4 The importance of alcohol prevention programs for work-related and commuting accidents

An accumulation of scientific evidence indicates that alcohol consumption—even in small amounts—significantly increases the risk of injuries and accidents both in the workplace and during commuting to and from work. As emphasized by Chikritzhs and Livingston, the relationship between alcohol consumption and injury risk is linear: higher levels of alcohol intake are directly associated with a greater likelihood of accident incidents, regardless of the type of work performed (50). From a public health perspective, this means that reducing alcohol consumption among the working population is one of the key components of injury and accident prevention strategies.

In their systematic literature review, Dyreborg et al. pointed out that the effectiveness of occupational safety prevention measures increases when interventions address both technical and behavioral factors (51). Alcohol prevention programs—when incorporated into broader workplace safety strategies—may, in this context, serve as a key tool for reducing the number of accidents resulting from decreased concentration, delayed reaction times, and impaired risk assessment associated with alcohol use.

Importantly, research by Hulls et al. shows that in male-dominated sectors—where the risk of accidents and alcohol consumption has traditionally remained at higher levels—the implementation of targeted health and prevention programs yields particularly significant results (52). The authors pointed out that these interventions not only reduce the frequency of accidents but also improve the overall level of wellbeing and safety culture within organizations.

The synthesis of the above findings indicates that alcohol prevention programs play a significant role in reducing the number of workplace and commuting accidents. Their effectiveness increases when they form part of an integrated organizational safety strategy based on education, psychological support, and consistent enforcement of sobriety policies.

It is important to emphasize that the significance of alcohol prevention programs extends far beyond reducing the number of workplace and commuting accidents. Research indicated that alcohol consumption contributes to a wide range of adverse health, psychosocial, and organizational outcomes, including absenteeism, reduced productivity, deteriorating workplace relationships, increased staff turnover, greater strain on the healthcare system, and negative effects on families and local communities (53). This makes prevention programs a critical component of public health and occupational safety strategy.

Consequently, alcohol prevention program implemented as part of an integrated organizational health and safety strategy have the potential to generate benefits at multiple levels—not only by reducing injuries and accidents but also by supporting public health, enhancing productivity, improving workforce stability, and promoting the overall psychosocial wellbeing of employees.

### 4.5 The importance of destigmatization and the role of new technologies

One of the key challenges remains destigmatizing the use of support, particularly among risky drinkers or those who do not disclose their problems for fear of the consequences. Prevention programs that offer online support and guarantee anonymity greatly increase the accessibility of interventions and effectively break down existing barriers to participation. The role of employers in this aspect is becoming increasingly important—the active promotion of health policies nowadays goes beyond the traditional remit of medical departments and acquires importance in the context of building a culture of health in the organization.

Moreover, modern tools such as mobile apps, remote consultations, or online training make it possible to reach those people who would not normally benefit from traditional forms of assistance. The individualization of interventions—for example, tailoring the program to gender, age, or job position—further intensifies the effectiveness of the prevention provided.

### 4.6 Barriers and threats to policy effectiveness

Deficiencies in clear guidelines and a lack of consistent enforcement of alcohol policies are often associated with higher levels of alcohol consumption among employees, particularly in occupational settings with easy access to alcohol or liberal social norms. In turn, factors such as job instability, low job satisfaction or chronic stress—if not adequately addressed by prevention programs—contribute to the emergence and exacerbation of alcohol problems.

### 4.7 Implementation and partners

Alcohol prevention in the workplace is a topic of considerable health, social, and economic importance. Implementing alcohol prevention programs requires the collaboration of multiple stakeholders, as alcohol misuse affects not only employees’ health but also workplace safety, productivity, and organizational culture. When implementing such programs, it is important to clearly define the roles and responsibilities of governmental and local authorities, healthcare institutions, employers, HR departments, and employee representatives. The importance of intersectoral cooperation should also be emphasized, along with the need to consider organizational and cultural determinants. A cooperation model that outlines potential partners and their possible roles and responsibilities is presented in Table 3.

**Table 3:** Model of intersectoral cooperation in implementing workplace alcohol prevention programs.

Partner	Main role	Key action	Benefits
Labor market sector: employers and HR departments	*Organizer* Initiators and implementers of internal activities	Implementation of alcohol prevention programs and anti-alcohol policies (e.g., prohibition of alcohol consumption at work, clear response procedures). Organization of training sessions for employees and managerial staff on recognizing alcohol-related problems. Collaboration with external experts (e.g., psychologists, addiction therapists). Provision of anonymous employee support services [e.g., hotlines, counseling, employee assistance programs (EAPs)].	Improved workplace safety and efficiency, reduced absenteeism and accidents.
Public and private healthcare institutions	*Expert* Medical and therapeutic experts	Conducting preventive screenings and health consultations. Developing and implementing therapeutic programs for individuals at risk of addiction. Cooperating with workplaces in conducting educational campaigns. Providing guidance on the early identification of problematic alcohol use symptoms.	Improvement of employees’ overall health and early intervention in case of risky behaviors.
Governmental and local authorities	*Regulator* Coordinators, regulators, and funding bodies	Developing legal frameworks and guidelines for employers regarding prevention initiatives. Financing or co-financing workplace prevention programs. Conducting educational campaigns. Supporting research and evaluation of prevention program effectiveness. Promoting best practices and certifying organizations implementing health policies. Monitoring and evaluating the effectiveness of prevention activities.	A comprehensive, system-level approach to prevention encompassing all sectors of the labor market.
Non-governmental organizations and professional associations	*Social support* Implementation partners and social advisors	Conducting workshops, campaigns, and training sessions. Developing educational materials. Providing anonymous support channels (e.g., support groups or helplines).	Strengthening the social and psychological dimension of prevention activities.
Labor unions and employee councils	*Employee advocate* Employee interest representatives	Co-developing and consulting on workplace anti-alcohol policies. Supporting employees in therapeutic or reintegration processes. Promoting a culture of safety and responsibility.	Increased acceptance and effectiveness of programs among employees.

Source: Authors’ own study based on Refs. (29, 46, 48, 82).

## 5 Conclusion

Effective alcohol prevention in the workplace requires the involvement of all organizational levels and should be regarded as an element of development strategy and an investment in human capital. The best results are achieved through comprehensive programs that combine education, training, clear procedures, the provision of anonymous support, and regular monitoring of outcomes. Integrated actions help reduce harmful alcohol consumption, improve employee wellbeing, decrease workplace accidents and injuries, reduce absenteeism, and thereby enhance overall organizational effectiveness.

## Footnotes

1 Disability-adjusted life years (DALYs)—a measure of overall disease burden, representing the sum of years of life lost due to premature mortality (YLL) and years lived with disability (YLD).

2 Available online at: https://www.parpa.pl/phocadownloadpap/Uzaleznienie/Wzory%20spozywania%20alkoholu.%20Leczenie%20osob%20uzaleznionych%20od%20alkoholu%20-%20broszura.pdf (Accessed May 6, 2025).

3 Available online at: https://www.who.int/health-topics/alcohol#tab=tab_1 (Accessed May 6, 2025).

4 Available online at: https://www.healthywork.org (Accessed May 6, 2025).

## References

[1] Rehm J Gmel GE Gmel G . The relationship between different dimensions of alcohol consumption and burden of disease-an update. Addiction. (2017) 112:968–1001. doi: 10.1111/add.13757 28220587 PMC5434904

[2] Nutt DJ King LA Phillips LD. Drug harms in the UK: a multicriteria decision analysis. Lancet. (2010) 376:1558–65. doi: 10.1016/S0140-6736(10)61462-6, 21036393

[3] American Psychiatric Association. Diagnostic and statistical manual of mental disorders (DSM-5). BMC Med. (2013) 17:133–7. doi: 10.1097/NMD.0b013e3182a2168a

[4] World Health Organization. Global status report on alcohol and health. Geneva: WHO (2018).

[5] Ryniak J Bętkowska-Korpała B. Aspekty psychoterapeutyczne uzależnienia od alkoholu (2013). Available online at: https://www.mp.pl/pacjent/psychiatria/uzaleznienia/81200,aspekty-psychoterapeutyczne-uzaleznienia-od-alkoholu (Accessed May 6, 2025).

[6] Rehm J Mathers C Popova S Thavorncharoensap M Teerawattananon Y Patra J. Global burden of disease and injury and economic cost attributable to alcohol use and alcohol-use disorders. Lancet. (2009) 373:2223–33. doi: 10.1016/S0140-6736(09)60746-7, 19560604

[7] Państwowa Agencja Rozwiązywania Problemów Alkoholowych. Wzory spożywania alkoholu. Leczenie osób uzależnionych od alkoholu- brochure. (n.d.). Available online at: https://www.parpa.pl/phocadownloadpap/Uzaleznienie/Wzory%20spozywania%20alkoholu.%20Leczenie%20osob%20uzaleznionych%20od%20alkoholu%20-%20broszura.pdf (Accessed May 6, 2025).

[8] Piotrowicz Z Ochwanowska E. Alkohol a układ nerwowy. Kosmos Probl Nauk Biol. (2012) 61:133–42.

[9] Gilpin NW Koob GF. Neurobiology of alcohol dependence: focus on motivational mechanisms. Alcohol Res Health. (2008) 31:185–95. 19881886 PMC2770186

[10] Kranzler HR Knapp C Ciraulo D. Pharmacotherapy of alcoholism In: Kranzler HR Ciraulo DA Zindel LR, editors. Psychopharmacology. 2nd ed. Washington, DC: American Psychiatric Publishing (2014). 1–69.

[11] Hillemacher T Leggio L Heberlein A. Studies of pharmacological treatments for alcoholism. Expert Opin Investig Drugs. (2015) 24:17–30. doi: 10.1517/13543784.2014.954037, 25164385 PMC12434415

[12] Salling MC. Using circuits to understand addiction In: Gilpin NW, editor. Neurobiology of addiction. Cambridge, MA: Academic Press (2023). 1–44.

[13] Kranzler HK. Overview of alcohol use disorder. Am J Psychiatry. (2023) 180:565–72. doi: 10.1176/appi.ajp.20230488, 37525595

[14] Amiri S Behnezhad S. Alcohol consumption and sick leave: a meta-analysis. J Addict Dis. (2020) 38:100–12. doi: 10.1080/10550887.2020.1724606, 32037988

[15] Hashemi N Skogen JC Sevic A . A systematic review and meta-analysis uncovering the relationship between alcohol consumption and sickness absence: when type of design, data, and sickness absence make a difference. PLoS One. (2022) 17:e0262458. doi: 10.1371/journal.pone.0262458 35015789 PMC8752011

[16] Schou L Moan IS. Alcohol use-sickness absence association and the moderating role of gender and socioeconomic status: a literature review. Drug Alcohol Rev. (2016) 35:158–69. doi: 10.1111/dar.12278, 26331574

[17] Frone MR. Predictors of overall and on-the-job substance use among young workers. J Occup Health Psychol. (2003) 8:39–54. doi: 10.1037/1076-8998.8.1.39, 12553528

[18] Thørrisen MM Skogen JC Aas RW. The associations between employees' risky drinking and sociodemographics, and implications for intervention needs. BMC Public Health. (2018) 18:735. doi: 10.1186/s12889-018-5660-x, 29898703 PMC6000943

[19] Marchand A Parent-Lamarche A Blanc M-È. Work and high-risk alcohol consumption in the Canadian workforce. Int J Environ Res Public Health. (2011) 8:2692–705. doi: 10.3390/ijerph8072692, 21845153 PMC3155324

[20] Skogen JC Bøe T Thørrisen MM Riper H Aas RW. Sociodemographic characteristics associated with alcohol consumption and alcohol-related consequences: a latent class analysis of the Norwegian WIRUS Screening Study. BMC Public Health. (2019) 19:1364. doi: 10.1186/s12889-019-7648-6, 31651277 PMC6814033

[21] Park S Lee J-H Lee J. Alcohol abuse associated with accumulated periods of precarious employment: a four-year follow-up study of a young population in Korea. Int J Environ Res Public Health. (2022) 19:7380. doi: 10.3390/ijerph19127380, 35742626 PMC9223578

[22] Mellibruda J Sobolewska-Mellibruda Z. Integrative psychotherapy of addiction: Theory and practice. Warsaw, Poland: IPZ PTP (2006).

[23] Szcześniak E. Terapia osób uzależnionych od alkoholu w podstawowym etapie leczenia. Studia Gdańskie Wizje i Rzeczywistość. (2011) 8:283–92.

[24] Frąckowiak M Motyka M. Alcohol dependence syndrome: characteristics, phases of addiction, methods of diagnosis. Probl Hig Epidemiol. (2015) 96:315–20.

[25] Mellibruda J. Alcoholism and the diagnosis of alcohol dependence. (n.d.). Available online at: http://www.psychologia.edu.pl/czytelnia/50-artykuly/1032-alkoholizm-i-diagnozowanie-uzaleznienia-od-alkoholu.html (Accessed May 6, 2025).

[26] Thørrisen MM Bonsaksen T Hashemi N Kjeken I van Mechelen W Aas RW. Association between alcohol consumption and impaired work performance (presenteeism): a systematic review. BMJ Open. (2019) 9:e029184. doi: 10.1136/bmjopen-2019-029184, 31315869 PMC6661906

[27] World Health Organization. Harmful use of alcohol. (n.d.). Available online at: https://www.who.int/health-topics/alcohol#tab=tab_1 (Accessed May 6, 2025).

[28] Yuvaraj K Eliyas SK Gokul S Manikandanesan S. Effectiveness of workplace intervention for reducing alcohol consumption: a systematic review and meta-analysis. Alcohol Alcohol. (2019) 54:264–71. doi: 10.1093/alcalc/agz024, 30957142

[29] Sunami T So R Ishii H Sadashima E Ueno T Yuzuriha T . A randomized controlled trial of the web-based drinking diary program for problem drinking in multi workplace settings. J Occup Health. (2022) 64:e12312. doi: 10.1002/1348-9585.12312, 35026038 PMC8757573

[30] Roman PM Blum TC. The workplace and alcohol problem prevention. Alcohol Res Health. (2002) 26:49–57. 12154651 PMC6683807

[31] Blake H Yildirim M Premakumar V Morris L Miller P Coffey F. Attitudes and current practice in alcohol screening, brief intervention, and referral for treatment among staff working in urgent and emergency settings: an open, cross-sectional international survey. PLoS One. (2023) 18:e0291573. doi: 10.1371/journal.pone.0291573, 37756359 PMC10529549

[32] Koffarnus MN Kablinger AS Kaplan BA Crill EM. Remotely administered incentive-based treatment for alcohol use disorder with participant-funded incentives is effective but less accessible to low-income participants. Exp Clin Psychopharmacol. (2021) 29:555–65. doi: 10.1037/pha0000503, 34110885 PMC8943847

[33] Reynolds GS Lehman WEK. Levels of substance use and willingness to use the employee assistance program. J Behav Health Serv Res. (2003) 30:238–48. doi: 10.1007/BF02289811, 12710376

[34] Taylor SE Brown JD. Illusion and well-being: a social psychological perspective on mental health. Psychol Bull. (1988) 103:193–210. doi: 10.1037/0033-2909.103.2.193, 3283814

[35] Ames GM Grube J Moore R. Social control and workplace drinking norms: a comparison of two organizational cultures. J Stud Alcohol Drugs. (2000) 61:203–19. doi: 10.15288/jsa.2000.61.203, 10757130

[36] Øvretveit J. Improvement leaders: what do they and should they do? A summary of a review of research. Qual Saf Health Care. (2010) 19:490–2. doi: 10.1136/qshc.2010.041772, 21127110

[37] Landsbergis P Dobson M LaMontagne A Choi B Schnall P Baker D. Occupational stress In: Levy BWD Baron S Sokas R, editors. Occupational and environmental health. 7th ed. Oxford: Oxford University Press (2017)

[38] Leka S Cox T eds. PRIMA-EF: Guidance on the European framework for psychosocial risk management. Geneva: WHO (2008). 10.1539/joh.o10010 21325735

[39] Imamura K Asai Y Watanabe K Tsutsumi A Shimazu A Inoue A . Effect of the National Stress Check Program on mental health among workers in Japan: a 1-year retrospective cohort study. J Occup Health. (2018) 60:298–306. doi: 10.1539/joh.2017-0314-OA, 29669966 PMC6078839

[40] Healthy Work Campaign. Available online at: https://www.healthywork.org (Accessed May 6, 2025).

[41] Webb G Shakeshaft A Sanson-Fisher R Havard A. A systematic review of workplace interventions for alcohol-related problems. Addiction. (2009) 104:365–77. doi: 10.1111/j.1360-0443.2008.02472.x, 19207344

[42] Liira H Verbeek JH Ruotsalainen JH Costa G. Workplace interventions for preventing job loss and other work-related outcomes in workers with alcohol misuse. Cochrane Database Syst Rev. (2016) 9:CD008879. doi: 10.1002/14651858.CD012344, 23152265

[43] Pidd K Kostadinov V Roche AM. Do workplace policies work? An examination of the relationship between alcohol and other drug policies and workers' substance use. Int J Drug Policy. (2016) 28:48–54. doi: 10.1016/j.drugpo.2015.08.017, 26410610

[44] Pidd K Roche AM. How effective is drug testing as a workplace safety strategy? A systematic review of the evidence. Accid Anal Prev. (2014) 71:154–65. doi: 10.1016/j.aap.2014.05.012, 24922614

[45] Hermansson U Helander A Brandt L Huss A Rönnberg S. Screening and brief intervention for risky alcohol consumption in the workplace: results of a 1-year randomised controlled study. Alcohol Alcohol. (2010) 45:252–7. doi: 10.1093/alcalc/agq021, 20406791

[46] Fellbaum L Mojzisch A Bielefeld L Benit N Soellner R. The effectiveness of workplace interventions for the prevention of alcohol use: a meta-analysis. Addiction. (2023) 118:2043–61. doi: 10.1111/add.16276, 37394719

[47] Alfred L Limmer M Cartwright S. An integrative literature review exploring the impact of alcohol workplace policies. Int J Workplace Health Manag. (2021) 14:87–110. doi: 10.1108/IJWHM-10-2019-0130, 35579975

[48] Morse AK Askovic M Sercombe J Dean K Fisher A Marel C . A systematic review of the efficacy, effectiveness and cost-effectiveness of workplace-based interventions for the prevention and treatment of problematic substance use. Front Public Health. (2022) 10:1051119. doi: 10.3389/fpubh.2022.1051119, 36419993 PMC9676969

[49] Elling DL Almquist YB Wennberg P Sundqvist K. Effects of a multi-component alcohol prevention program in the workplace on hazardous alcohol use among employees. BMC Public Health. (2023) 23:1420. doi: 10.1186/s12889-023-16150-4, 37488547 PMC10367231

[50] Chikritzhs T Livingston M. Alcohol and the risk of injury. Nutrients. (2021) 13:2777. doi: 10.3390/nu13082777, 34444939 PMC8401155

[51] Dyreborg J Lipscomb HJ Nielsen K Törner M Rasmussen K Frydendall KB . Safety interventions for the prevention of accidents at work: a systematic review. Campbell Syst Rev. (2022) 18:e1234. doi: 10.1002/cl2.1234, 36911341 PMC9159701

[52] Hulls PM Richmond RC Martin RM Chavez-Ugalde Y de Vocht F. Workplace interventions that aim to improve employee health and well-being in male-dominated industries: a systematic review. Occup Environ Med. (2022) 79:77–87. doi: 10.1136/oemed-2020-107314, 34035181 PMC8785069

[53] Popescu C Burlea Ș Diaconu L . Alcohol consumption in the workplace: a comparison between European Union countries’ policies. Int J Environ Res Public Health. (2022) 19:16964. doi: 10.3390/ijerph192416964 36554848 PMC9779578

[54] Thørrisen MM Skogen JC Bonsaksen T Skarpaas LS Aas RW. Are workplace factors associated with employee alcohol use? The WIRUS cross-sectional study. BMJ Open. (2022) 12:e064352. doi: 10.1136/bmjopen-2022-064352, 36229146 PMC9562323

[55] Jonsson J Muntaner C Bodin T Alderling M Rebeka R Burström B . Low-quality employment trajectories and risk of common mental disorders, substance use disorders and suicide attempt: a longitudinal study of the Swedish workforce. Scand J Work Environ Health. (2021) 47:509–20. doi: 10.5271/sjweh.3978, 34397098 PMC8504160

[56] El Haddad R Matta J Lemogne C . The association between substance use and subsequent employment among students: prospective findings from the CONSTANCES cohort. Soc Psychiatry Psychiatr Epidemiol. (2022) 57:2335–45. doi: 10.1007/s00127-022-02357-0 PMC9437401 36053312

[57] Baek SU Yoon JH Won JU. Investigating the potential association of temporary employment and job dissatisfaction with alcohol use disorder and depressive symptoms: a 13-wave longitudinal analysis. BJPsych Open. (2023) 9:e115. doi: 10.1192/bjo.2023.40, 37041110 PMC10134250

[58] Murray K Murphy C Herlihy A . Harmful alcohol consumption in elite sports players in Ireland. Ir J Med Sci. (2021) 190:1491–8. doi: 10.1007/s11845-021-02819-5 PMC8545772 34699001

[59] Mou NL Lei WK Balaji S Contractor AA Latkin CA Hall BJ. The association between posttraumatic disorder symptoms and addictive behaviours in Macao within a sample of female Filipino migrant workers: a network analysis. Eur J Psychotraumatol. (2023) 14:2178764. doi: 10.1080/20008066.2023.2178764, 37052088 PMC9987736

[60] Thern E Elling DL Badarin K . Precarious employment in young adulthood and later alcohol-related morbidity: a register-based cohort study. Occup Environ Med. (2023) 80:213–21. doi: 10.1136/oemed-2023-109315 PMC11103336 38627100

[61] Grandey AA Frone MR Melloy RC Sayre GM. When are fakers also drinkers? A self-control view of emotional labor and alcohol consumption among US service workers. J Occup Health Psychol. (2019) 24:236–51. doi: 10.1037/ocp0000147, 30829513 PMC6675660

[62] Azagba S Shan L Wolfson M Hall M Chaloupka F. Problem drinking as intentional risky behaviour: examining the association between state health insurance coverage and excessive alcohol consumption. Prev Med Rep. (2021) 24:101556. doi: 10.1016/j.pmedr.2021.101556, 34976624 PMC8683933

[63] Kim JY Kim J Park S Fear N. Workplace victimization and alcohol misuse among junior military personnel: mediating the role of anger. J Affect Disord. (2021) 295:855–62. doi: 10.1016/j.jad.2021.07.010, 34332364

[64] Hsu S-T Wu H-C Chien H-T Li D-J. Predictors of workplace substance reuse among patients with alcohol or illegal substance use disorder in the workplace. Int J Environ Res Public Health. (2022) 19:10023. doi: 10.3390/ijerph191610023, 36011658 PMC9408551

[65] Oesterle S Bailey JA Catalano RF . Alcohol-tolerant workplace environments are a risk factor for young adult alcohol misuse on and off the job in Australia and the United States. Int J Environ Res Public Health. (2021) 18:6725. doi: 10.3390/ijerph20186725 37754585 PMC10530761

[66] Bonsaksen T Thørrisen MM Skogen JC Hesse M Aas RW. Are demanding job situations associated with alcohol-related presenteeism? The WIRUS-screening study. Int J Environ Res Public Health. (2021) 18:6169. doi: 10.3390/ijerph18116169, 34200397 PMC8201186

[67] Cannizzaro E Cirrincione L Malta G Fruscione S Mucci N Martines F . The influence of the COVID-19 pandemic emergency on alcohol use: a focus on a cohort of Sicilian workers. Int J Environ Res Public Health. (2023) 20:4613. doi: 10.3390/ijerph20054613, 36901622 PMC10001951

[68] McKetta S Prins SJ Hasin D Patrick ME Keyes KM. Structural sexism moderates work and occupational risks for alcohol consumption and binge drinking among US women, 1989-2016. Soc Sci Med. (2023) 324:115878. doi: 10.1016/j.socscimed.2023.115878, 37003025 PMC10121897

[69] Lee M Lee U Park J-H . The association between alcohol use and suicidal ideation among employees. Psychiatry Investig. (2021) 18:808–15. doi: 10.30773/pi.2021.0122 PMC8542748 34525778

[70] Thern E Blindow KJ Jonsson E . Hazardous alcohol consumption across different industries in Sweden: a pooled cross-sectional study. Alcohol Alcohol. (2023) 58:agac077. doi: 10.1093/alcalc/agac077 PMC11554268 39527838

[71] Rospenda KM Richman JA McGinley M Moilanen KL Lin T Johnson TP . Effects of chronic workplace harassment on mental health and alcohol misuse: a long-term follow-up. BMC Public Health. (2023) 23:1430. doi: 10.1186/s12889-023-16219-0, 37495970 PMC10373226

[72] Baek SU Won JU Yoon JH. Association between long working hours and the onset of problematic alcohol use in young workers: a population-based longitudinal analysis in South Korea. J Affect Disord. (2023) 344:141–8. doi: 10.1016/j.jad.2023.10.020, 37820956

[73] Thørrisen MM Bonsaksen T Skogen JC Skarpaas LS Sevic A van Mechelen W . Willingness to participate in alcohol prevention interventions targeting risky drinking employees. The WIRUS project. Front Public Health. (2021) 9:692605. doi: 10.3389/fpubh.2021.692605, 34249850 PMC8267363

[74] Berge LI Gjestad R Franck J Javaras KN Greenfield S Haver B. Gender specific early treatment for women with alcohol addiction (EWA): impact on work related outcomes. A 25-year registry follow-up of a randomized controlled trial (RCT). Drug Alcohol Depend. (2022) 239:109600. doi: 10.1016/j.drugalcdep.2022.109600, 36007448 PMC9509484

[75] Elling DL Almquist YB Wennberg P Sundqvist K. Evaluation of a workplace alcohol prevention program targeted on managers' inclination to initiate early alcohol intervention. Work. (2022) 73:517–26. doi: 10.3233/WOR-210943, 35938276 PMC9661318

[76] Manning V Piercy H Garfield JBB Clark SG Andrabi MN Lubman DI. A personalised approach bias modification smartphone app ("SWiPE") to reduce alcohol use: open-label feasibility, acceptability, and preliminary effectiveness study. JMIR Mhealth Uhealth. (2021) 9:e31353. doi: 10.2196/31353, 34890355 PMC8709909

[77] Lubman DI Grigg J Reynolds J . Effectiveness of a stand-alone telephone-delivered intervention for reducing problem alcohol use. Exp Clin Psychopharmacol. (2021) 29:555–65. doi: 10.1001/jamapsychiatry.2022.2779 36129698 PMC9494267

[78] Barticevic NA Poblete F Zuzulich SM Rodriguez V Bradshaw L. Brief motivational therapy versus enhanced usual care for alcohol use disorders in primary care in Chile: study protocol for an exploratory randomized trial. Trials. (2020) 21:692. doi: 10.1186/s13063-020-04589-4, 32736578 PMC7393703

[79] Kelly JF Humphreys K Ferri M. Alcoholics anonymous and other 12-step programs for alcohol use disorder. Cochrane Database Syst Rev. (2020) 2020:CD012880. doi: 10.1002/14651858.CD012880.pub2, 32159228 PMC7065341

[80] Garnett C Perski O Beard E Michie S West R Brown J. The impact of celebrity influence and national media coverage on users of an alcohol reduction app: a natural experiment. BMC Public Health. (2021) 21:30. doi: 10.1186/s12889-020-10011-0, 33407283 PMC7789329

[81] Traxler HK Kaplan BA Rzeszutek MJ Franck CT Koffarnus MN. Interest in and perceived effectiveness of contingency management among alcohol drinkers using behavioral economic purchase tasks. Exp Clin Psychopharmacol. (2022) 31:127–39. doi: 10.1037/pha0000580, 35708948 PMC10103538

[82] Łukowska K. Rozwiązywanie problemów alkoholowych w społecznościach lokalnych. Poradnik dla praktyków. Warszawa: Mazowieckie Centrum Polityki Społecznej; 2019.

